# Patterns of multimorbidity and polypharmacy in young and adult population: Systematic associations among chronic diseases and drugs using factor analysis

**DOI:** 10.1371/journal.pone.0210701

**Published:** 2019-02-06

**Authors:** Enrica Menditto, Antonio Gimeno Miguel, Aida Moreno Juste, Beatriz Poblador Plou, Mercedes Aza Pascual-Salcedo, Valentina Orlando, Francisca González Rubio, Alexandra Prados Torres

**Affiliations:** 1 CIRFF, Center of Pharmacoeconomics, University of Naples Federico II, Naples, Italy; 2 Aragon Health Sciences Institute (IACS), IIS Aragón, REDISSEC ISCIII, Miguel Servet University Hospital, Zaragoza, Spain; 3 Aragon Health Service (SALUD), Zaragoza, Spain; Universidad de Malaga, SPAIN

## Abstract

**Objectives:**

The objective was to identify the systematic associations among chronic diseases and drugs in the form of patterns and to describe and clinically interpret the constituted patterns with a focus on exploring the existence of potential drug-drug and drug-disease interactions and prescribing cascades.

**Methods:**

This observational, cross-sectional study used the demographic and clinical information from electronic medical databases and the pharmacy billing records of all users of the public health system of the Spanish region of Aragon in 2015. An exploratory factor analysis was conducted based on the tetra-choric correlations among the diagnoses of chronic diseases and the dispensed drugs in 887,572 patients aged ≤65 years. The analysis was stratified by age and sex. To name the constituted patterns, assess their clinical nature, and identify potential interactions among diseases and drugs, the associations found in each pattern were independently reviewed by two pharmacists and two doctors and tested against the literature and the information reported in the technical medicinal forms.

**Results:**

Six multimorbidity-polypharmacy patterns were found in this large-scale population study, named as respiratory, mental health, cardiometabolic, endocrinological, osteometabolic, and mechanical-pain. The nature of the patterns in terms of diseases and drugs differed by sex and age and became more complex as age advanced.

**Conclusions:**

The six clinically sound multimorbidity-polypharmacy patterns described in this non-elderly population confirmed the existence of systematic associations among chronic diseases and medications, and revealed some unexpected associations suggesting the prescribing cascade phenomenon as a potential underlying factor. These findings may help to broaden the focus and orient the early identification of potential interactions when caring for multimorbid patients at high risk of adverse health outcomes due to polypharmacy.

## Introduction

Optimization of drug prescribing is emerging as a mandatory element for healthcare systems [1]. Prescribing is largely based on single-disease evidence-based guidelines, which do not generally consider chronic multimorbidity (i.e., co-occurrence of several chronic diseases within a patient). Consequently, patients are prescribed several drugs following multiple disease-specific guidelines [2].

The resulting polypharmacy, defined as the use of multiple medicines, is not always appropriate. Several studies have shown that inappropriate polypharmacy increases the risk of unnecessary drug use, potential drug-drug and drug-disease interactions, and adverse drug reactions (ADRs) [3–5]. Polypharmacy is often due to the so-called ‘prescribing cascade’, which involves the clinician’s failure to recognize a new medical event as an ADR. In such cases, an additional drug is prescribed to treat the adverse reaction leading to side effects instead of withdrawing or changing the responsible drug, thus creating a vicious circle and adding further risks to multimorbid patients [6–7].

Large-scale population studies aiming to explore real-life patterns of polypharmacy represent a unique opportunity to analyse the complexity of drug prescribing, and explore the existence of systematic associations among drugs. A recent study identified several polypharmacy patterns in a large population, and their clinical interpretation suggested the existence of underlying causal factors that were often related, not to the disease itself, but to the side effects of the prescribed treatments. The study highlighted the need for analyses combining diseases and drugs, as both can be causal and consequent factors of inappropriate drug prescription [8]. Although the burden of chronic diseases and drugs prescribed, and subsequently the risk of interactions among them, increases with age, this problem is not exclusive to the elderly, and research should also focus on younger populations to allow the early identification of potential interactions and the development of prevention strategies.

This large-scale population study aimed to characterize the existence of systematic associations among chronic diseases and drugs in the form of patterns in young and adult populations and to describe and clinically interpret the constituted patterns with a focus on exploring the existence of potential drug-drug and drug-disease interactions and prescribing cascades.

## Materials and methods

### Study design, data sources, and study population

We conducted a cross-sectional, observational study in the EpiChron Cohort [9] using data from 2015. This cohort integrates anonymized demographic, clinical and drug dispensation information of all users of the public health system in Aragon, a region of north-eastern Spain (1,144,816 inhabitants in 2015).

Patients aged >65 years were excluded from the study to allow focus on young and adult populations. Furthermore, preliminary tests conducted in the elderly revealed that a high number of diseases and drugs present multicollinearity (i.e., linear correlation), leading to the creation of a singular data matrix that invalidates the use of factor analysis. The study population included 887,572 patients, who were stratified into three age groups: 0–14, 15–44, and 45–65 years.

We considered demographic variables (i.e., age and sex), diagnoses of chronic diseases from primary care and hospitals, and dispensed drugs during 2015 from pharmacy billing records. Diagnoses were originally coded according to the International Classification of Primary Care (ICPC) and to the International Classification of Diseases, 9^th^ Revision (ICD-9), and were grouped in the Expanded Diagnostic Clusters (EDC) of the Johns Hopkins ACG System (version 11.0, The Johns Hopkins University, Baltimore, MD, US). All 114 diseases classified as chronic by Salisbury et al [10] were included in the analysis and coded in binary format (i.e., absence/presence of the disease). Additionally, we included rhinitis, according to the recent World Health Organization (WHO) indications [11], and acute lower respiratory tract infection, as it can lead to chronic sequelae. Drugs were coded according to the Anatomical Therapeutic Chemical Classification (ATC) System at the third level to facilitate data processing, also in binary format.

The study was approved by the Clinical Research Ethics Committee of Aragon (CEICA), which waived the requirement for patient consent since data of the EpiChron Cohort are anonymized, and no interventions on individuals were performed.

### Statistical analysis

A descriptive analysis of the population was performed by calculating the frequencies of chronic conditions and drugs dispensed in each sex and age group.

Multimorbidity and polypharmacy patterns were identified using exploratory factor analysis based on a correlation matrix to determine which diagnoses and dispensed drugs comprised each pattern. This technique was previously used to cluster chronic conditions [12] and medications [8] separately. We used tetra-choric correlation matrices due to the dichotomous nature of both chronic diagnoses and dispensed drugs. Factor extraction was performed using the principal factor method. An oblique rotation (Oblimin) was applied to facilitate factor interpretation. We used scree plots to determine the number of factors to be extracted in each group. When a clear solution was not obtained by the scree plot, a clinical evaluation of different solutions was conducted by EM, FGR, and MAPS. To determine which EDCs and ATC codes formed each pattern we selected those with scores ≥0.30 for each factor, which is the threshold factor loading traditionally used when deciding wheter to accept a variable as belonging to a factor [13]. EDCs and ATC codes with scores from 0.25–0.30 were included in a factor if considered relevant and useful in the clinical explanation of the pattern [8]. The factors resulting from this analysis were interpreted as multimorbidity and polypharmacy patterns.

To increase the epidemiological interest of the study, we included in the analysis only EDCs with a prevalence ≥1–2% in each age and sex group. Likewise, ATC codes with a prevalence ≥3–5% in each subgroup were considered for analysis. Some ATCs with lower prevalence were also included based on their potential relevance for interactions or side effects. In contrast, several ATC codes presented multicollinearity with specific EDCs and were discarded to allow statistical analysis. In these cases, ATC codes were manually excluded in the order of the degree of multicollinearity until the factor analysis gave satisfactory results. The list of dispensed drugs was reviewed by two pharmacists (EM, MAPS) and one general practitioner (FGR). Final inclusion and exclusion criteria of EDCs and ATC codes are specified for each sex and age group.

In children aged 0–14 years, EDCs with a prevalence ≥1% and ATC codes with a prevalence ≥3% were included, except for vitamins A and D, including combinations of the two. Propulsives, decongestants and antiallergics, psychostimulants, agents used for attention-deficit hyperactivity disorder (ADHD) and nootropics, drugs for peptic ulcer and gastro-esophageal reflux disease, and antiepileptics, were also included regardless of their prevalence based on their potential to cause ADRs.

In patients aged 15–44 years, EDCs with a prevalence ≥2% and ATC codes with a prevalence ≥3% were included, except for lipid modifying agents due to collinearity with disorders of lipid metabolism. Antiepileptics, antipsychotics, corticosteroids for systemic use, thyroid preparations, opioids, anti-inflammatory agents and anti-infective in combination, antithrombotic agents, and antimigraine preparations, were included regardless of their prevalence.

For women aged 45–65 years, EDCs with a prevalence ≥2% and ATC codes with a prevalence ≥5% were included, except for lipid modifying agents, thyroid preparations and iron preparations, due to collinearity with disorders of lipoid metabolism, thyroid disease, iron deficiency and other deficiency anaemias, respectively. For men aged 45–65 years, the same inclusion criteria were used, but lipid modifying agents, blood glucose lowering drugs excluding insulins, angiotensin-converting enzyme inhibitors, combinations of angiotensin II antagonist, beta blocking agents, and antigout preparations, were excluded due to collinearity with disorders of lipoid metabolism, diabetes, hypertension, and gout.

Sample adequacy was evaluated using the Kaiser-Meyer-Olkin (KMO) test. Only values >0.60 were considered as acceptable. Additionally, as a measure of the model’s goodness-of-fit, we calculated the proportion of cumulative variance, which describes the data variability explained by the patterns. All statistical analyses were conducted in STATA (Version 12.0, StataCorp LLC, College Sation, TX, US).

### Denomination and clinical nature of the patterns

To assess the clinical nature of the patterns identified by statistical criteria, and to identify potential interactions among diseases and drugs within the patterns, three consecutive steps were followed. First, the associations found in each pattern were independently reviewed by two pharmacists (EM and MAPS) and two doctors (FGR and APT) from the research team and with proven expertise to look for potential inappropriate medication, prescribing cascade, and drug-drug, drug-disease, and disease-disease interactions. Second, a consensus meeting was held to discuss and resolve discrepancies and to name the patterns based on their clinical nature. Third, the findings were tested against the literature and the information reported in the technical medicinal forms.

## Results

The mean number of concomitant diseases increased with age, from one condition registered in children aged 0–14 years to almost three conditions in adults aged 45–65 years (Table 1). The number of dispensed drugs followed the same trend and increased from two medications in children to almost four dispensations in adults aged 45–65.

**Table 1:** Mean number of chronic diseases^a^ and dispensed drugs^b^ according to age and sex groups.

		Women	Men	Total
**0–14 years**	N (%)	78,534 (8.85)	82,893 (9.34)	161,427 (18.2)
	Chronic diseases (95% CI)	1.00 (1.00–1.01)	1.12 (1.11–1.12)	1.06 (1.05–1.07)
	Dispensed drugs (95% CI)	2.16 (2.15–2.18)	2.27 (2.26–2.29)	2.22 (2.21–2.23)
**15–44 years**	N (%)	205,122 (23.1)	190,658 (21.5)	395,780 (44.6)
	Chronic diseases (95% CI)	1.47 (1.46–1.47)	1.14 (1.14–1.15)	1.31 (1.31–1.32)
	Dispensed drugs (95% CI)	2.67 (2.66–2.68)	1.78 (1.77–1.78)	2.24 (2.23–2.25)
**45–65 years**	N (%)	168,587 (19.0)	161,778 (18.2)	330,365 (37.2)
	Chronic diseases (95% CI)	3.06 (3.04–3.07)	2.48 (2.47–2.49)	2.77 (2.76–2.78)
	Dispensed drugs (95% CI)	4.34 (4.32–4.36)	3.42 (3.41–3.44)	3.89 (3.88–3.90)
**Total**	N (%)	452,243 (51.0)	435,329 (49.0)	**887,572 (100)**
	Chronic diseases (95% CI)	1.98 (1.97–1.98)	1.63 (1.63–1.64)	1.81 (1.81–1.81)
	Dispensed drugs (95% CI)	3.20 (3.19–3.21)	2.48 (2.47–2.49)	2.85 (2.84–2.86)

**Abbreviations**: CI, confidence interval; N, number of patients.

^a^ According to Salisbury et al.; rhinitis and acute lower respiratory tract infection were also included.

^b^ ATC (Anatomical Therapeutic Chemical Classification) codes at the third level.

### Multimorbidity and polypharmacy patterns

Six different patterns of multimorbidity and polypharmacy were identified in the study population, named respiratory, mental health, cardiometabolic, endocrinological, osteometabolic, and mechanical pain, according to their clinical nature. Respiratory, mental health, and cardiometabolic patterns occurred in both men and women. Endocrinological and osteometabolic patterns appeared only in women, whereas the mechanical pain pattern appeared exclusively in men (Table 2). Three different variants were described in the respiratory pattern: a generic one, a pattern with acute infection, and a respiratory pattern with an asthma-allergic component. The nature of the patterns in terms of diseases and drugs differed depending on sex and age. The patterns found in each age and sex group are described below. The scree plots are reported in S1 and S2 Figs and the factor scores in S1 Table.

**Table 2:** Multimorbidity and polypharmacy patterns identified in each age and sex group.

	0–14 years	15–44 years	45–65 years
**Women**	Respiratory-acute infection	Mental health	Mental health
	Respiratory-asthma-allergic	Respiratory	Respiratory
	Mental health	Endocrinological	Cardiometabolic
			Osteometabolic
**Men**	Respiratory-acute infection	Mental health	Mental health
	Respiratory-asthma-allergic	Mechanical pain	Cardiometabolic
	Mental health	Respiratory	Respiratory

#### Boys aged 0–14 years

This age and sex group had a KMO sampling adequacy index of 0.740. The proportion of cumulative variance explained by the patterns was 0.356. The scree plot and clinical evaluation indicated the extraction of three factors (Table 3). Factor 1 clustered acute respiratory infection and a pharmacological pattern for the symptomatic treatment with corticoids, inhaled beta-adrenergic agonists, antipyretics, antihistamines, and nonsteroidal anti-inflammatory drugs (NSAIDS). Antibiotics (i.e., macrolides and penicillin antibiotics) could be present to treat the potential bacterial superinfection [8]. The potential therapeutic cascades identified in this pattern were: a) antifungal drugs for the treatment of candidiasis secondary to antibiotics [8] and corticoids inhalers [14]; b) electrolytes for the treatment of gastroenteritis dehydration after use of antibiotics [14]; and c) anxiolytics, supposedly prescribed for the symptomatic treatment of the potential side effects of adrenergic inhalants (e.g., tachycardia, hyperactivity, and insomnia) dispensed for the symptomatic treatment of acute respiratory infection [14]. As potential drug-drug interactions (DDIs) we observed the combined use of NSAIDs and corticoids [15].

**Table 3:** Patterns of chronic diseases (EDC codes) and drugs (ATC codes) and loading factor scores in boys aged 0–14 years. Diseases are highlighted in bold.

EDC/ATC	Disease/Drug	Factor 1	Factor 2	Factor 3
H02A	Corticosteroids for systemic use, pain	0.6877		
**RES02**	**Acute lower respiratory tract infection**	0.6748		
R03A	Adrenergics, inhalants	0.6683	0.3420	
J01C	Beta-lactam antibacterials, penicillins	0.5854		
R03B	Other drugs for obstructive airway diseases, inhalants	0.5520	0.4091	
N02B	Other analgesics and antipyretics	0.5332		
J01F	Macrolides, lincosamides and streptogramins	0.5120		
N05B	Anxiolytics	0.4556		
S01A	Anti-infective	0.4545		
D07A	Corticosteroids, plain	0.4018		
M01A	Anti-inflammatory and antirheumatic products, non-steroids	0.3990		
A07C	Electrolytes with carbohydrates	0.3666		
D01A	Antifungals for topical use	0.3452		
D06A	Antibiotics for topical use	0.3344		
R06A	Antihistamines for systemic use	0.3143	0.6159	
ALL03	Allergic rhinitis		0.7213	
S01G	Decongestants and antiallergics		0.6773	
R01A	Decongestants and other nasal preparations for topical use		0.6734	
**ASMA**	**Asthma**		0.4222	
N06B	Psychostimulants, agents used for ADHD and nootropics			0.7213
N03A	Antiepileptics			0.6562
**PSY05**	**Attention deficit disorder**			0.5889
**PSY14**	**Psychosocial disorders of childhood**			0.3968
**NUR19**	**Developmental disorder**			0.3857
A02B	Drugs for peptic ulcers and GERD			0.3324

**Abbreviations**: ADHD, attention deficit hyperactivity disorder; ATC, Anatomical Therapeutic Chemical Classification; EDC, Expanded Diagnostic Clusters; GERD, gastro-esophageal reflux disease.

**Notes**: Kaiser-Meyer-Olkin (KMO): 0.740; % of cumulative variance explained: 35.6%.

Factor 2 clustered a respiratory-allergic pattern comprising asthma and allergic rhinitis with medications such as antihistamines, antiallergics, decongestants, other nasal preparations for topical use and beta-adrenergic agonists.

Factor 3 clustered developmental, psychosocial disorders and ADHD to drugs for the treatment of these diseases, such as psychostimulants, agents used for ADHD and antiepileptics. This pattern also included an unexpected association with proton pump inhibitors (PPIs), which are drugs for the treatment of peptic ulcers. PPIs might have been used to prevent upper gastrointestinal tract bleeding or gastroesophageal reflux disease due to the use of antidepressants [8,16,17]. Potential DDIs observed in this pattern were: a) the combined use of carbamazepine and methylphenidate [14]; and b) carbamazepine and omeprazole [18].

#### Men aged 15–44 years

This age and sex group had a KMO sampling adequacy index of 0.751. The proportion of cumulative variance explained by the patterns was 0.370. The scree plot for this group indicated that the number of factors to be extracted was equal to three (Table 4). Factor 1 clustered psychopathological processes (e.g., depression, anxiety, sleep disorders, psychosis, and neurosis and substance use) and drugs including antidepressants, anxiolytics, antiepileptics and antipsychotics. A potential interaction identified in this pattern was substance abuse, including alcohol consumption which represents a potential risk for DDIs with psychotropic medication, resulting in sedation and drowsiness [19].

**Table 4:** Patterns of chronic diseases (EDC codes) and drugs (ATC codes) and factor loading scores in men aged 15–44 years. Diseases are highlighted in bold.

EDC/ATC	Disease/Drug	Factor 1	Factor 2	Factor 3
N06A	Antidepressants	0.8979		
N05C	Hypnotics and sedatives	0.7614		
N05A	Antipsychotics	0.7482		
N05B	Anxiolytics	0.6522		
N03A	Antiepileptics	0.6442		
**PSY09**	**Depression**	0.6005		
**PSY02**	**Substance use**	0.4973		
**PSY01**	**Anxiety neuroses**	0.4801		
**PSY19**	**Sleep disorders of nonorganic origin**	0.4604		
M01A	Anti-inflammatory and antirheumatic products, non-steroids		0.7741	
N02B	Other analgesics and antipyretics		0.6115	
A02B	Drugs for peptic ulcers and GERD		0.5996	
J01C	Beta-lactam antibacterials, penicillins		0.5105	
N02A	Opioids		0.4920	
**MUS14**	**Low back pain**		0.4663	
H02A	Corticosteroids for systemic use, pain		0.4642	
J01F	Macrolides, Lincosamides, and streptogramins		0.4037	
B01A	Antithrombotic agents		0.3980	
**RES02**	**Acute lower respiratory tract infection**		0.3072	0.3838
R03A	Adrenergics, inhalants			0.7900
R06A	Antihistamines for systemic use			0.7005
**ASMA**	**Asthma**			0.6227
R01A	Decongestants and other nasal preparations for topical use			0.5562
**ALL03**	**Allergic rhinitis**			0.4093

**Abbreviations**: ATC, Anatomical Therapeutic Chemical Classification; EDC, Expanded Diagnostic Clusters; GERD, gastro-esophageal reflux disease.

**Notes**: Kaiser-Meyer-Olkin (KMO): 0.751; % of cumulative variance explained: 37.0%.

Factor 2 clustered a wide range of medications used for the treatment of chronic pain, such as opioids, corticosteroids, analgesics, antipyretics, and anti-inflammatories. This pattern was unexpectedly associated with: a) antithrombotic agents comprising both heparins and acetylsalicylic acid, typically used for the prevention of thromboembolism after surgery and/or long-term stays (which can be caused by musculoskeletal pain); b) drugs for peptic ulcers, probably prescribed to treat the gastrointestinal side effects of antithrombotics, analgesics, and corticosteroids [15,18,20]; and c) macrolides, penicillin antibiotics, and drugs for peptic ulcers. The potential DDIs identified in this pattern were: a) the interaction of fentanyl with macrolides, which increases the effect of the opioid and the risk of respiratory depression [14]; b) acetylsalicylic acid with diclofenac [21]; c) omeprazole with warfarin [18]; and d) omeprazole and esomeprazole with clopidogrel [15].

Factor 3 showed a respiratory pattern with a chronic allergic component. This factor clustered acute respiratory infection, allergic-rhinitis and asthma, and medications such as antihistamines, antiallergics, decongestants, other nasal preparations for topical use and beta-adrenergic agonists.

#### Men aged 45–65 years

This age and sex group had a KMO sampling adequacy index of 0.627. The proportion of cumulative variance explained by the patterns was 0.304. The scree plot for this group suggested extracting four factors. However, the Heywood phenomenon occurred, and the clinical evaluation finally recommended extracting three factors (Table 5).

**Table 5:** Patterns of chronic diseases (EDC codes) and drugs (ATC codes) and factor loading scores in men aged 45–65 years. Diseases are highlighted in bold.

EDC/ATC	Disease/Drug	Factor 1	Factor 2	Factor 3
N06A	Antidepressants	0.7887		
N05B	Anxiolytics	0.7326		
N03A	Antiepileptics	0.6613		
**PSY09**	**Depression**	0.5530		
N02A	Opioids	0.4891		
**PSY01**	**Anxiety, neuroses**	0.4447		
M01A	Anti-inflammatory and antirheumatic products, non-steroids	0.4166		
A02B	Drugs for peptic ulcers and GERD	0.3990	0.3952	
**PSY19**	**Sleep disorders of nonorganic origin**	0.3594		
**MUS14**	**Low back pain**	0.3367		
**MUS13**	**Cervical pain syndromes**	0.3161		
N02B	Other analgesics and antipyretics	0.3113		0.3056
**NUR21**	**Neurologic disorders, other**	0.2959		
B01A	Antithrombotic agents		0.7832	
**HTA**	**Hypertension**		0.6610	
**IHD**	**Ischemic heart disease**		0.6085	
**DIAB**	**Diabetes**		0.5750	
C09C	Angiotensin II antagonists, plain		0.5396	
CAR16	Cardiovascular disorders, other		0.4854	
**CAR09**	**Cardiac arrhythmia**		0.4723	
**NUT03**	**Obesity**		0.4283	
**RES04**	**Emphysema, chronic bronchitis, COPD**		0.3380	0.3491
**CAR11**	**Disorders of lipid metabolism**		0.3296	
**RHU02**	**Gout**		0.3014	
R03A	Adrenergics, inhalants			0.8130
R06A	Antihistamines for systemic use			0.7063
**RES02**	**Acute lower respiratory tract infection**			0.5897
R01A	Decongestants and other nasal preparations for topical use			0.5803
**ASMA**	**Asthma**			0.5666
J01M	Quinolone antibacterials			0.4548
J01F	Macrolides, lincosamides, and streptogramins			0.4383
J01C	Beta-lactam antibacterials, penicillins			0.3981
**ALL03**	**Allergic rhinitis**			0.3589

**Abbreviations**: ATC, Anatomical Therapeutic Chemical Classification; COPD, chronic obstructive pulmonary disease; EDC, Expanded Diagnostic Clusters; GERD, gastro-esophageal reflux disease.

**Notes**: Kaiser-Meyer-Olkin (KMO): 0.627; % of cumulative variance explained: 30.4%. The scree plot for this group suggested extracting 4 factors. However, the Heywood phenomenon occurred, and the clinical evaluation finally recommended extracting 3 factors.

The first pattern identified was very similar to Factor 1 found in younger men, but a neurological component and a pain component (i.e., lower back pain) were also present. However, substance use was no longer present. Several DDIs were identified in this pattern: a) the use of carbamazepine as antiepileptic drug and omeprazole [18]; b) the dispensation of antidepressants and drugs for neuropathic pain [16]; and c) the combined use of benzodiazepines with PPIs and opioids, which could increase sedation [15].

Factor 2 was determined by the association among hypertension, diabetes, obesity, disorders of lipid metabolism and complex cardiovascular disorders (e.g., cardiac arrhythmia and ischaemic disease) and drugs for the treatment of these conditions. This factor also included chronic obstructive pulmonary disease (COPD).

Factor 3 was very similar to that found in younger men but also included emphysema, chronic bronchitis, and COPD and antibiotics (e.g., macrolides, quinolone, and penicillin). The potential DDI identified was the use of macrolides with inhaled beta-adrenergic and antihistamines, producing a QT prolongation and thus increasing the risk of arrhythmia [14].

#### Girls aged 0–14 years

This age and sex group had a KMO sampling adequacy index of 0.732. The proportion of cumulative variance explained by the patterns was 0.332. The scree plot and clinical discussion recommended the extraction of three factors (Table 6). The resulting patterns were similar to Factor 1, Factor 2 and Factor 3 identified in boys aged 0–14 years. However, beta-adrenergic agonists and other nasal preparations for topical use were absent in Factor 2, and Factor 3 did not comprise ADHD, which is more frequent in men than in women at this age [22].

**Table 6:** Patterns of chronic diseases (EDC codes) and drugs (ATC codes) and factor loading scores in girls aged 0–14 years. Diseases are highlighted in bold.

EDC/ATC	Disease/Drug	Factor 1	Factor 2	Factor 3
H02A	Corticosteroids for systemic use, pain	0.6427		
**RES02**	**Acute lower respiratory tract infection**	**0.6355**		
R03A	Adrenergics, inhalants	0.6224		
J01C	Beta-lactam antibacterials, penicillins	0.5882		
N02B	Other analgesics and antipyretics	0.5116		
J01F	Macrolides, Lincosamides, and streptogramins	0.4816		
N05B	Anxiolytics	0.457		
S01A	Anti-infectives	0.4271		
M01A	Anti-inflammatory and antirheumatic products, non-steroids	0.4174		
D07A	Corticosteroids, plain	0.4097		
D01A	Antifungals for topical use	0.3684		
A07C	Electrolytes with carbohydrates	0.3648		
D06A	Antibiotics for topical use	0.3583		
R06A	Antihistamines for systemic use	0.3299	0.6105	
**ALL03**	**Allergic rhinitis**		0.7546	
S01G	Antihistamines for systemic use		0.7419	
R01A	Decongestants and antiallergics		0.6744	
**ASMA**	**Asthma**		0.3489	
N03A	Antiepileptics			0.6693
N06B	Psychostimulants, agents used for ADHD and nootropics			0.5403
**NUR19**	**Developmental disorder**			0.3793
A02B	Drugs for peptic ulcers and GERD			0.3761
**PSY14**	**Psychosocial disorders of childhood**			0.3287

**Abbreviations**: ADHD, attention deficit hyperactivity disorder; ATC, Anatomical Therapeutic Chemical Classification; EDC, Expanded Diagnostic Clusters; GERD, gastro-esophageal reflux disease.

**Notes**: Kaiser-Meyer-Olkin (KMO): 0.732; % of cumulative variance explained: 33.2%.

#### Women aged 15–44 years

This age and sex group had a KMO sampling adequacy index of 0.720. The proportion of cumulative variance explained by the patterns was 0.299. The scree plot and clinical discussion indicated that the number of factors extracted was equal to three (Table 7). The first factor was similar to Factor 1 identified in men of the same age, but this pattern also comprised neurological disorders and peripheral neuropathy in women, as well as other drugs including opioids, antimigraine drugs, NSAIDs and drugs for peptic ulcers and gastro-esophageal reflux disease (GERD). The presence of opioids was unexpected in this pattern and could cause a number of DDIs because of combined use with selective serotonin reuptake inhibitors (SSRIs), a type of antidepressant that increases the risk of serotonin syndrome, which in turn increases the risk of convulsions [23].

**Table 7:** Patterns of chronic diseases (EDC codes) and drugs (ATC codes) and factor loading scores in women aged 15–44 years. Diseases are highlighted in bold.

EDC/ATC	Disease/Drug	Factor 1	Factor 2	Factor 3
N06A	Antidepressants	0.8600		
N03A	Antiepileptics	0.7610		
N05B	Anxiolytics	0.7584		
N05A	Antipsychotics	0.5738		
**PSY09**	**Depression**	0.5535		
A02B	Drugs for peptic ulcers and GERD	0.4688		
N02A	Opioids	0.4575		
**PSY01**	**Anxiety, neuroses**	0.4333		
**PSY19**	**Sleep disorders of nonorganic origin**	0.3776		
N02C	Antimigraine preparations	0.3742		
**NUR21**	**Neurologic disorders, other**	0.3556		
M01A	Anti-inflammatory and antirheumatic products, non-steroids	0.3550	0.3224	
**NUR03**	**Peripheral neuropathy, neuritis**	0.3093		
R06A	Antihistamines for systemic use		0.8167	
R03A	Adrenergics, inhalants		0.7087	
R01A	Decongestants and other nasal reparations for topical use		0.6800	
S01G	Decongestants and antiallergics		0.6329	
**ASMA**	**Asthma**		0.4935	
**RES02**	**Acute lower respiratory tract infection**		0.4617	
**ALL03**	**Allergic rhinitis**		0.4243	
H02A	Corticosteroids for systemic use, plain		0.4065	
J01F	Macrolides, lincosamides and streptogramins		0.3837	
J01C	Beta-lactam antibacterials, penicillins		0.3651	
J01M	Anti-inflammatory and antirheumatic products, non-steroids		0.3413	
J01D	Quinolone antibacterials		0.3320	
N02B	Other beta-lactam antibacterials		0.3169	
D07A	Other analgesics and antipyretics		0.3086	
B03A	Iron preparations			0.7959
**H03C**	**Iodine therapy**			0.6469
**HEM02**	**Iron deficiency, other deficiency anemias**			0.5369
B03B	Vitamin B12 and folic acid			0.4798
H03A	Thyroid preparations			0.4306
**END04**	**Hypothyroidism**			0.3658

**Abbreviations**: ATC, Anatomical Therapeutic Chemical Classification; EDC, Expanded Diagnostic Clusters; GERD, gastro-esophageal reflux disease.

**Notes**: Kaiser-Meyer-Olkin (KMO): 0.740; % of cumulative variance explained: 35.6%.

Factor 2 clustered acute respiratory infection, allergic-rhinitis and asthma with medications such as corticoids, inhaled beta-adrenergic agonists, antipyretics, antihistamines, NSAIDS, quinolones, macrolides, and other beta-lactam antibacterials. The DDIs identified were: a) the use of inhaled beta-adrenergic agonists and corticosteroids, which decrease potassium levels, thus increasing the risk of arrhythmia [15]; and b) the combined use of macrolides and inhaled beta-adrenergic and antihistamines, producing a QT prolongation and thus increasing the risk of arrhythmia [14].

Factor 3 clustered hypothyroidism and iron and other deficiency anaemias. Drugs related to this pattern were thyroid hormone, iron therapy, iodine preparations, vitamin B12, and folic acid. Thyroid hormone was used for hypothyroidism treatment. The presence of iron preparations and vitamin B12 might be attributable to the treatment of autoimmune hypothyroidism produced by their deficiency in such patients [24–25].

#### Women aged 45–65 years

This age and sex group had a KMO sampling adequacy index of 0.803. The proportion of cumulative variance explained by the patterns was 0.313. The scree plot for this group indicated the extraction of four factors (Table 8). The first factor was similar to Factor 1 observed in younger women but without the neurological component. The second factor was also similar to Factor 2 observed in younger women with the absence of some medications, such as NSAIDs. Factor 3 clustered a typical metabolic syndrome with hypertension, diabetes, obesity, and lipid metabolism disorders. The drugs related to this pattern were antithrombotic agents for cardiovascular prevention and ACE inhibitors for hypertension treatment [8]. The absence of antihyperlipidaemic drugs was due to the collinearity observed between antihyperlipidaemics and lipid metabolism disorders, which required their exclusion from the analysis due to statistical needs. Factor 4 comprised osteoporosis and calcium therapy.

**Table 8:** Patterns of chronic diseases (EDC codes) and drugs (ATC codes) and factor loading scores in women aged 45–65 years. Diseases are highlighted in bold.

EDC/ATC	Disease/Drug	Factor 1	Factor 2	Factor 3	Factor 4
N06A	Antidepressants	0.8980			
N05B	Anxiolytics	0.6682			
**PSY09**	**Depression**	0.6131			
N05C	Hypnotics and sedatives	0.5592			
N03A	Antiepileptics	0.5406			
**PSY01**	**Anxiety, neuroses**	0.4116			
N02A	Opioids	0.3805			
**PSY19**	**Sleep disorders of nonorganic origin**	0.3618			
A02B	Drugs for peptic ulcers and GERD	0.3379			
R03A	Adrenergics, inhalants		0.7548		
R06A	Antihistamines for systemic se		0.7487		
R01A	Decongestants and other nasal preparations for topical use		0.6301		
**ASMA**	**Asthma**		0.5872		
H02A	Corticosteroids for systemic use, pain		0.4867		
J01F	Macrolides, lincosamides and streptogramins		0.4468		
J01M	Quinolone antibacterials		0.4313		
**ALL03**	**Allergic rhinitis**		0.4032		
J01C	Beta-lactam antibacterials, penicillins		0.3853		
N02B	Other analgesics and antipyretics		0.3269		
**HTA**	**Hypertension**			0.9601	
C09A	ACE inhibitors, plain			0.7041	
**DIAB**	**Diabetes**			0.5854	
**NUT03**	**Obesity**			0.5014	
B01A	Antithrombotic agents			0.3699	
**CAR11**	**Disorders of lipid metabolism**			0.2951	
A12A	Calcium				0.8032
**END02**	**Osteoporosis**				0.7869

**Abbreviations**: ATC, Anatomical Therapeutic Chemical Classification; EDC, Expanded Diagnostic Clusters; GERD, gastro-esophageal reflux disease.

**Notes**: Kaiser-Meyer-Olkin (KMO): 0.803; % of cumulative variance explained: 31.3%.

## Discussion

### Main findings

A total of six clinically relevant patterns of multimorbidity and polypharmacy were found in the young and adult population of the study, named respiratory, mental health, cardiometabolic, endocrinological, osteometabolic, and mechanical pain. Differences found in their composition depended in part on the sex of the patient and all patterns became more complex as age increased.

The respiratory pattern was present in all age and sex groups. It comprised a group of drugs administered for the same category of diseases, including medications that were used to treat complications of these illnesses (e.g., topical antifungal agents, electrolytes) or the side effects of other drugs (e.g., anxiolytics). In the 15–44 and 45–65 age subgroups, acute-infection and rhinitis and asthma merged in a respiratory pattern showing a chronic-allergic component in both men and women. The associated medication pattern associated showed the addition of quinolones, probably for infection exacerbation. In men aged 15–44 and 45–65, the use of corticoids did not appear in the pattern, but we cannot confirm that they were not prescribed. Some therapeutic absences in women aged 45–65 years are worth noting such as NSAIDs, which could be under prescribed because of the risk of digestive and cardiovascular side effects [8] and antifungals for topical use, most likely due to the lower incidence of vaginal candidiasis in postmenopausal women [8]. Treatment differences between men and women emerging from the analysis should be further investigated.

Mental health pattern was present in all age and sex groups, varying considerably between groups, and became more complex as age increased. In men aged 15–44 years, the mental health pattern comprised neither neurological disorders nor peripheral neuropathy, NSAIDs, opioids, or PPIs. The pattern included psychopathological processes such as depression, anxiety, sleep disorders, psychosis, and neurosis, which are likely related to substance use, also present in this pattern, which commonly affects men in this age range, as already reported by Prados-Torres et al. [12]. Substance abuse includes alcohol consumption, which represents a potential risk for DDIs with psychotropic medication, resulting in sedation and drowsiness [19]. The neuro-psychiatric pattern in this sex and age group could thus be due to substance abuse. Other diseases could also be consequence of some type of dependency. This causal hypothesis is supported by the fact that this pattern did not appear in women, in which toxic substance use occurs less frequently, as supported by the bibliography [12].

A consistent cardiometabolic pattern is described in our study, with a composition already reported in the bibliography [12]. This pattern appeared only in men and women aged 45–65 years. In women, this pattern describes a typical metabolic syndrome. In men, other associated conditions were detected, such as COPD, gout and complex cardiovascular disorders, possibly due to increased cardiovascular risk in men, together with increased incidence of ischemic heart and cerebrovascular diseases [8]. The presence of emphysema, chronic bronchitis, and COPD can be related to the association between the cardiac and respiratory domains already described and supported in the literature [20]. The presence of antithrombotic agents in men could be related to the prevention of cardiovascular diseases and to the treatment of ischemic disease and arrhythmia. The use of PPIs is widely recommended for patients taking antiplatelet agents [8]. However, we did not find PPIs in women treated with antithrombotic agents, which was unexpected and cannot be further explained. Indeed, the absence of PPIs was already observed in a previous polypharmacy cluster analysis performed in our study cohort [8]. On the other hand, the use of different antihypertensive drugs in men and women was not justified from a clinical point of view [26]. It is worth highlighting the different pharmacological approaches followed in middle-aged men and women in our study, as the use of different antihypertensive drugs cannot be supported scientifically [26–27].

In men aged 15–44 years, the presence of a mechanical-pain pattern was evident. Mino-León et al. [20] recently observed that the associations among the vascular, upper gastrointestinal, and musculoskeletal domains could be a consequence of two factors. First, changes that occur in the connective tissue with ageing, have been linked to a low grade of inflammation. Second, side effects are related to the pharmacological treatment of the diseases included in the musculoskeletal pattern [20], such as the treatment of gastrointestinal disease caused by NSAIDs, corticosteroids, and antithrombotic agents [15,18,20] It is noteworthy that the pattern also comprised macrolides, penicillin antibiotics, and drugs for peptic ulcers, all of which are usually used for the eradication of *Helicobacter pylori* (*H*. *pylori*). Indeed, NSAID-naïve users should be tested for the presence of *H*. *pylori* infection and, if positive, receive eradication therapy before NSAID use, a practice that is well accepted and supported by strong evidence [28].

In women aged 15–44 and 45–65 years we identified an endocrinological and an osteometabolic pattern, respectively. The presence of iron and other deficiency anaemia observed in women aged 15–44 years could be due to conditions such as menstruation and pregnancy in which the use of the abovementioned preparations is more necessary. The dispensation of calcium appeared alone in the osteometabolic pattern. The combined use of calcium and other osteoporosis treatments shows certain protective effect for the prevention of hip and non-vertebral fractures. Although the use of Vitamin D is recommended for osteoporosis, this medication did not appear in this pattern, which is in agreement with the current deficiency reported for this vitamin [29].

Several examples of potential DDIs at increased risk of adverse health outcomes were observed in our study, including potential DDIs in the respiratory pattern for the different age subgroups, such as: a) the use of inhaled beta-adrenergic agonists and corticosteroids, which decreased potassium levels, thus increasing the risk of arrhythmia [15]; b) the use of macrolides with inhaled beta-adrenergic and antihistamines, producing a QT prolongation and thus increasing the risk of arrhythmia [14]; and c) the combined use of NSAIDs and corticoids in the 0–14 age subgroup, which can increase the gastrointestinal risk [15]. In the mental health pattern, we also reported DDIs such as: a) the combined use of carbamazepine for epilepsy and methylphenidate for the treatment of ADHD which may decrease the effect of methylphenidate [14]; c) antidepressants and drugs for neuropathic pain [16]; and d) the combined use of benzodiazepines with PPIs and opioids, which increases sedation [15].

Other associations described in the present study cannot be fully rationalized and should be further investigated as they may give new clues to a better understanding of the relationship between multimorbidity and polypharmacy.

### Strengths and limitations

To our knowledge, this is the first large-scale population study exploring the systematic association among chronic diseases and dispensed drugs. The large population size of the EpiChron Cohort, which has already served as a basis for several pharmaco-epidemiological studies [30–32], together with the quality of data, resulted in reliable and representative results compared to those based only on medical records or drug use surveys. Furthermore, the goodness-of-fit values of the obtained models (i.e., KMO sampling adequacy index and proportion of cumulative variance explained) indicated that factor analysis is an appropriate statistical technique to achieve the aims of the study in the target population [12].

However, one of the most important methodological limitations of this study concerns the impossibility of including some drugs in the analyses due to multicollinearity with specific diseases, thus leading to the absence of specific drugs that would be, a priori, expected in some patterns. The issue of multicollinearity was also responsible for excluding the population aged >65 years from the analysis, which limited the comprehensiveness of the study. Further investigations using complementary methodological approaches are needed to identify the systematic association among chronic diseases and drugs in the elderly, in which potential interactions among drugs and diseases would be more relevant due to the higher burden of chronic diseases and medications.

Several hypotheses have been arisen regarding the clinical explanation that underlies the six multimorbidity and polypharmacy patterns revealed in this study. However, they must be interpreted with caution since the study design (i.e., cross-sectional) does not allow the establishment of the sequence in which diseases and medications cluster within a pattern. Longitudinal studies would be necessary to corroborate the suggested causal associations and elucidate the associations that could not be explained in the present study. Another limitation of the study stems from the lack of information on the use of over-the-counter (OTC) medications, which could lead to underestimation of the actual drug use.

### Comparison with other studies

In 2012, a study based on exploratory factor analysis conducted in patients over 14 years old supported the existence of mechanical-obesity, metabolic, neurovascular, liver disease, psychiatric-substance abuse, anxiety, and depression-related patterns [12]. These results largely coincide with our findings and support the existence of the multimorbidity patterns described. The main difference with our study is that we analysed multimorbidity only in populations aged up to 65 years, similar to two Spanish studies conducted with information obtained from electronic medical records and the primary care pharmacy database in 2008 [8,12]. One of the studies stablished the existence of multimorbidity patterns [12] and the other one demonstrated the existence of non-random associations in drug prescription, resulting in patterns of polypharmacy [8].

The present study can be considered more exhaustive because it connects multimorbidity and polypharmacy patterns and evidences the existence of some unexpected systematic associations among chronic diseases and drugs, as well as potential DDIs and prescribing cascades described in multimorbid patients.

### Implications for health systems

This study validates part of the results obtained from a previous factor analysis study exploring associations between drugs [8]. The discovery of non-random associations among drugs and diseases could help in the development and/or adaptation of clinical guidelines to chronic patients with multimorbidity who are taking multiple drugs. Understanding the way in which drugs are associated with multimorbidity will bring about a better understanding of polypharmacy management allowing us to better identify inappropriate polypharmacy. This has been urgently requested by the scientific community [33–34].

## Conclusion

This study revealed the existence of systematic associations among chronic diseases and dispensed drugs in both men and women up to 65 years of age, showing that they may occur at all ages, including children, and that they have a lifelong evolution. Six patterns of multimorbidity and polypharmacy were identified, named respiratory, mental health, cardiometabolic, endocrinological, osteometabolic, and mechanical-pain. The clinical interpretation of such patterns suggests that, apart from some expected associations related to the pharmacological treatment of diseases, the existence of drug-drug interactions and prescribing cascades may be a potential underlying factor for some of the associations identified among chronic diseases and drugs. The evidence of unexpected systematic associations between diseases and drugs in the patterns may help in the early identification of potential interactions in multimorbid patients with a high risk of adverse health outcomes due to polypharmacy.

## Supporting information

S1 Fig Scree plots for three age groups in women. (TIF)

S2 Fig Scree plots for three age groups in men. (TIF)

S1 Table Factor scores. This file contains S1A–S1F Tables. S1A Table, Factor scores for women aged 0–14 years. S1B Table, Factor scores for women aged 14–44 years. S1C Table, Factor scores for women aged 45–65 years. S1D Table, Factor scores for men aged 0–14 years. S1E Table, Factor scores for men aged 14–44 years. S1F Table, Factor scores for men aged 45–65 years.
(PDF)
