# Supplementary figures and images for: Patterns of multimorbidity and polypharmacy in young and adult population: Systematic associations among chronic diseases and drugs using factor analysis
Source: PLoS One. 2019 Feb 6;14(2):e0210701. doi: 10.1371/journal.pone.0210701 (PMC6364882; doi:10.1371/journal.pone.0210701)

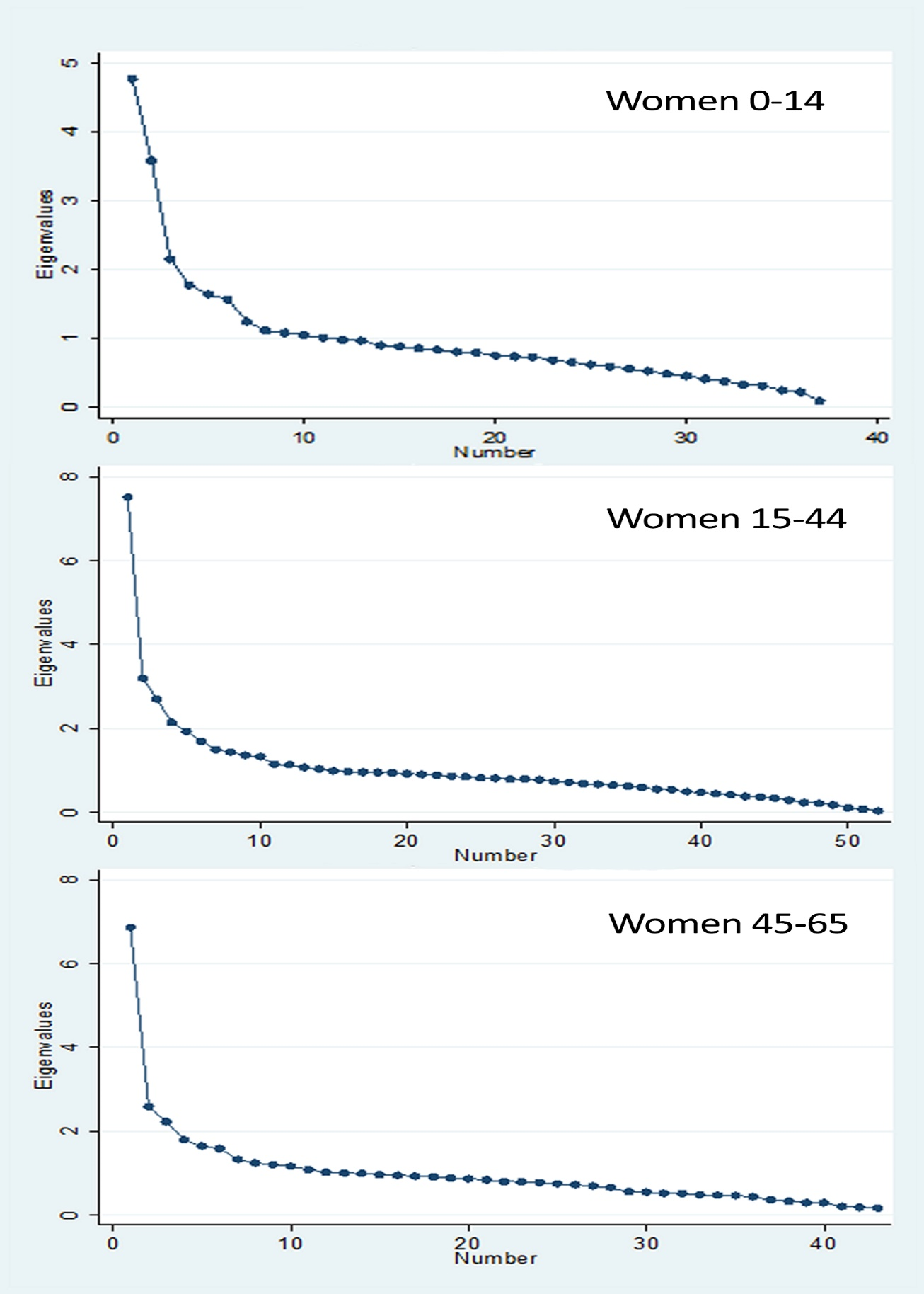

Supplement: S1 Fig — (TIF) [file pone.0210701.s001.tif]

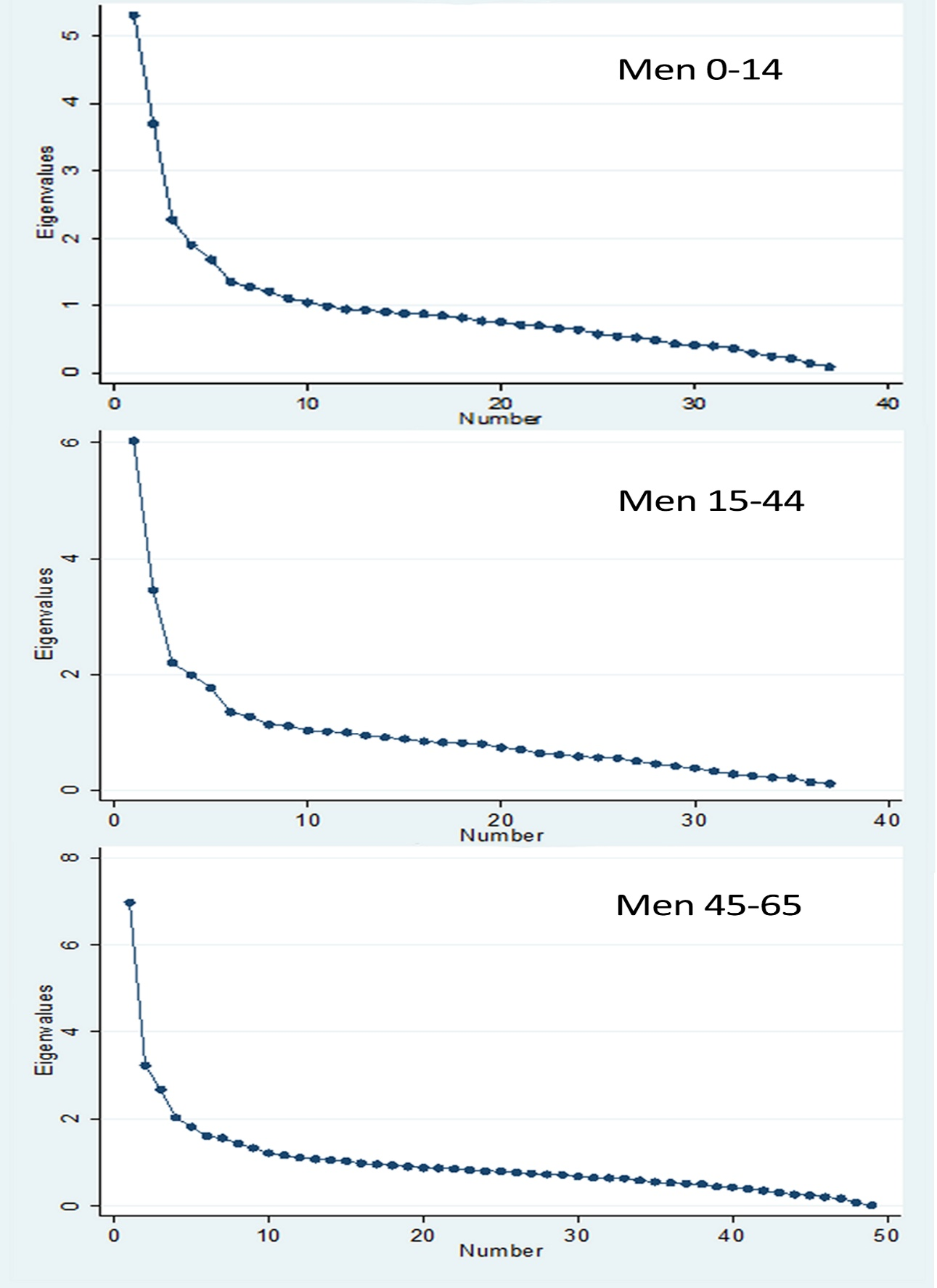

Supplement: S2 Fig — (TIF) [file pone.0210701.s002.tif]
