# Supplementary material for: Patterns of multimorbidity and polypharmacy in young and adult population: Systematic associations among chronic diseases and drugs using factor analysis
Source: PLoS One. 2019 Feb 6;14(2):e0210701. doi: 10.1371/journal.pone.0210701 (PMC6364882; doi:10.1371/journal.pone.0210701)
Supplement: S1 Table — This file contains S1A–S1F Tables. S1A Table, Factor scores for women aged 0–14 years. S1B Table, Factor scores for women aged 14–44 years. S1C Table, Factor scores for women aged 45–65 years. S1D Table, Factor scores for men aged 0–14 years. S1E Table, Factor scores for men aged 14–44 years. S1F Table, Factor scores for men aged 45–65 years. (PDF) [file pone.0210701.s003.pdf]

S1 Table

S1A Table - Factor scores for men aged 0-14 years

ATC/EDC	Drug/Disease	Factor1	Factor2	Factor3
H02A	Corticosteroids for systemic use, pain	0.6877	0.1341	-0.1872
RES02	Acute lower respiratory tract infection	0.6748	0.1509	-0.3011
R03A	Adrenergics, inhalants	0.6683	0.3420	-0.2071
J01C	Beta-lactam antibacterials, penicillins	0.5854	-0.0037	0.0007
R03B	Other drugs for obstructive airway diseases, inhalants	0.5520	0.4091	-0.0897
N02B	Other analgesics and antipyretics	0.5332	-0.1136	0.0209
J01F	Macrolides, lincosamides and streptogramins	0.5120	0.1248	0.0228
N05B	Anxiolytics	0.4556	-0.1763	0.2344
S01A	Anti-infective	0.4545	-0.0615	-0.2738
D07A	Corticosteroids, plain	0.4018	0.0069	-0.0309
M01A	Anti-inflammatory and antirheumatic products, non-steroids	0.3990	0.0946	0.1732
A07C	Electrolytes with carbohydrates	0.3666	-0.1027	0.0051
D01A	Antifungals for topical use	0.3452	-0.0277	-0.0462
D06A	Antibiotics for topical use	0.3344	-0.0364	0.0310
R06A	Antihistamines for systemic use	0.3143	0.6159	-0.0569
A03F	Propulsives	0.2743	0.0308	0.1180
ALL03	Allergic rhinitis	-0.1412	0.7213	0.0881
S01G	Decongestants and antiallergics	0.0161	0.6773	-0.0039
R01A	Decongestants and other nasal preparations for topical use	0.0733	0.6734	0.1450
ASMA	Asthma	0.1195	0.4222	0.0276
N06B	Psychostimulants, agents used for ADHD and nootropics	-0.1011	-0.0886	0.7213
N03A	Antiepileptics	0.2486	-0.2065	0.6562
PSY05	Attention deficit disorder	-0.1135	-0.0152	0.5889
PSY14	Psychosocial disorders of childhood	-0.0978	0.0685	0.3968
NUR19	Developmental disorder	0.0614	-0.0954	0.3857
A02B	Drugs for peptic ulcers and GORD	0.2044	0.0917	0.3324
EYE02	Blindness	-0.1713	0.1316	0.2622
CAR11	Disorders of lipid metabolism	-0.0821	0.1629	0.2528
NUT03	Obesity	-0.0867	0.0898	0.2230
MUS06	Kyphoscoliosis	-0.2040	0.1324	0.2182
EAR08	Deafness, hearing loss	-0.0262	0.1059	0.1928
EAR09	Chronic pharyngitis and tonsillitis	-0.0143	0.1793	0.1793
PSY19	Sleep disorders of nonorganic origin	0.1529	-0.0038	0.1527
SKN13	Disease of hair and hair follicles	-0.0201	0.0466	0.1449
MUS11	Congenital anomalies of limbs, hands, and feet	0.0517	0.0364	0.0834
S02A	Antiinfectives (Otologicals)	0.2286	0.0328	-0.0018
SKN02	Dermatitis and eczema	0.2327	0.0643	-0.0320

Abbreviations: ADHD, attention deficit hyperactivity disorder; ATC, Anatomical Therapeutic Chemical Classification; EDC, Expanded Diagnostic Clusters; GORD, gastro-esophageal reflux disease.
Notes: Kaiser-Meyer-Olkin (KMO): 0.740; % cumulative variance: 35.6%.

S1B Table-Factor scores for men aged 15-44 years

ATC/EDC	Drug/Disease	Factor1	Factor2	Factor3
N06A	Antidepressants	0.8979	-0.0787	0.0592
N05C	Hypnotics and sedatives	0.7614	-0.0049	0.0222
N05A	Antipsychotics	0.7482	-0.0917	0.0194
N05B	Anxiolytics	0.6522	0.2206	-0.0163
N03A	Antiepileptics	0.6442	0.1661	-0.0639
PSY09	Depression	0.6005	-0.0855	0.0363
PSY02	Substance use	0.4973	-0.0842	-0.0242
PSY01	Anxiety neuroses	0.4801	-0.0516	0.0375
PSY19	Sleep disorders of nonorganic origin	0.4604	0.0063	0.0306
M01A	Anti-inflammatory and antirheumatic products, non-steroids	-0.0838	0.7741	-0.0858
N02B	Other analgesics and antipyretics	-0.0211	0.6115	0.0362
A02B	Drugs for peptic ulcers and GORD	0.1944	0.5996	-0.0663
J01C	Beta-lactam antibacterials, penicillins	-0.1109	0.5105	0.1106
N02A	Opioids	0.1518	0.4920	-0.0543
MUS14	Low back pain	0.0243	0.4663	-0.1528
H02A	Corticosteroids for systemic use, pain	0.0515	0.4642	0.2213
J01F	Macrolides, Lincosamides, and streptogramins	-0.0621	0.4037	0.2091
B01A	Antithrombotic agents	0.0422	0.3980	-0.0959
RES02	Acute lower respiratory tract infection	0.0290	0.3072	0.3838
R03A	Adrenergics, inhalants	0.0254	0.1636	0.7900
R06A	Antihistamines for systemic use	-0.0601	0.2746	0.7005
ASMA	Asthma	-0.0182	-0.0280	0.6227
R01A	Decongestants and other nasal preparations for topical use	-0.0641	0.2498	0.5562
ALL03	Allergic rhinitis	-0.0299	-0.0022	0.4093
SKN02	Dermatitis and eczema	0.0261	0.0863	0.1800
D07A	Corticosteroids, plain	0.0472	0.2393	0.1740
D01A	Antifungicals for dermatological use	0.0570	0.1946	0.1207
MUS06	Kyphoscoliosis	-0.0618	-0.0524	0.1172
EYE02	Blindness	0.0090	-0.0235	0.0742
SKN13	Disease of hair and hair follicles	0.0259	-0.0336	0.0653
EAR08	Deafness, hearing loss	0.0656	0.0849	0.0242
SKN12	Psoriasis	0.0523	0.0783	0.0202
NUT03	Obesity	0.1659	0.1174	0.0091
H03A	Thyroid preparations	0.1801	0.0907	0.0064
ARTRITIS	Arthritis	0.0537	0.1622	-0.0111
CAR11	Disorders of lipid metabolism	0.2167	0.0994	-0.0738
HTA	Hypertension	0.1899	0.1596	-0.0864

Abbreviations: ATC, Anatomical Therapeutic Chemical Classification; EDC, Expanded Diagnostic Clusters; GORD, gastro-esophageal reflux disease.
Notes: Kaiser-Meyer-Olkin (KMO): 0.751; % cumulative variance: 37.0%.

S1 C Table- Factor scores for men aged 45-65 years

ATC/EDC	Drug/Disease	Factor1	Factor2	Factor3
N06A	Antidepressants	0.7887	-0.0482	-0.1452
N05B	Anxiolytics	0.7326	-0.0315	-0.0516
N03A	Antiepileptics	0.6613	0.0178	-0.0737
PSY09	Depression	0.5530	-0.0149	-0.1063
N02A	Opioids	0.4891	0.0671	0.1071
PSY01	Anxiety, neuroses	0.4447	-0.0565	-0.0504
M01A	Anti-inflammatory and antirheumatic products, non-steroids	0.4166	-0.0804	0.2415
A02B	Drugs for peptic ulcersand GORD	0.3990	0.3952	0.1015
PSY19	Sleep disorders of nonorganic origin	0.3594	0.0316	0.0258
MUS14	Low back pain	0.3367	-0.1079	0.0926
MUS13	Cervical pain syndromes	0.3161	-0.0193	0.0446
N02B	Other analgesics and antipyretics	0.3113	0.1196	0.3056
NUR21	Neurologic disorders, other	0.2959	0.0990	-0.0022
PSY02	Substance use	0.2574	0.1332	-0.0972
NUR03	Peripheral neuropathy, neuritis	0.2547	0.0911	0.0389
B01A	Antithrombotic agents	0.0728	0.7832	-0.1041
HTA	Hypertension	-0.0895	0.6610	-0.0564
IHD	Ischemic heart disease	0.0429	0.6085	-0.1058
DIAB	Diabetes	-0.0416	0.5750	-0.0885
C09C	Angiotensin II antagonists, plain	-0.0902	0.5396	-0.0283
CAR16	Cardiovascular disorders, other	-0.0011	0.4854	0.0163
CAR09	Cardiac arrhythmia	-0.0130	0.4723	0.0045
NUT03	Obesity	-0.0349	0.4283	0.0331
RES04	Emphysema, chronic bronchitis, COPD	-0.0153	0.3380	0.3491
CAR11	Disorders of lipid metabolism	0.0451	0.3296	-0.0274
RHU02	Gout	-0.0556	0.3014	0.0003
EYE06	Cataract, aphakia	0.0090	0.2582	0.0282
R03A	Adrenergics, inhalants	-0.1538	0.1695	0.8130
R06A	Antihistamines for systemic use	0.0095	-0.0688	0.7063
RES02	Acute lower respiratory tract infection	-0.0477	0.1589	0.5897
R01A	Decongestants and other nasal preparations for topical use	0.0107	-0.0934	0.5803
ASMA	Asthma	-0.1164	-0.0707	0.5666
J01M	Quinolone antibacterials	0.0378	0.1862	0.4548
J01F	Macrolides, lincosamides, and streptogramins	0.0648	0.0226	0.4383
J01C	Beta-lactam antibacterials, penicillins	0.1058	0.0706	0.3981
ALL03	Allergic rhinitis	0.0443	-0.1708	0.3589
D07A	Corticoesteroids, plain	0.1729	0.0647	0.2294
SKN02	Dermatitis and eczema	0.1679	0.0159	0.1436
SKN12	Psoriasis	0.0351	0.0803	0.0777
ARTRITIS	Arthritis	0.1488	0.1996	0.0761
GUR04	Prostatic hypertrophy	0.1129	0.2071	0.0727
EAR08	Deafness, hearing loss	0.1129	0.0501	0.0698
GSU08	Varicose veins of lower extremities	0.0743	0.1562	0.0673
CANCER	Cancer	0.1061	0.1823	0.0524
EYE02	Blindness	0.1031	0.0110	0.0456

END05	Other endocrine disorders	0.1117	0.1575	0.0359
EYE08	Glaucoma	0.0051	0.1753	0.0330
END04	Hypothyroidism	0.0993	0.1468	0.0321
GUR09	Renal calculi	0.0566	0.1045	0.0239

Abbreviations: ATC, Anatomical Therapeutic Chemical Classification; COPD, chronic obstructive pulmonary disease; EDC, Expanded Diagnostic Clusters; GORD, gastro-esophageal reflux disease.
Notes: Kaiser-Meyer-Olkin (KMO): 0.627; % cumulative variance: 30.4%.

S1D Table- Factor scores for women aged 0-14 years

ATC/EDC	Drug/Disease	Factor1	Factor2	Factor3
H02A	Corticosteroids for systemic use, pain	0.6427	0.0923	-0.2782
RES02	Acute lower respiratory tract infection	0.6355	0.0858	-0.4024
R03A	Adrenergics, inhalants	0.6224	0.2459	-0.3373
J01C	Beta-lactam antibacterials, penicillins	0.5882	0.0297	0.0212
N02B	Other analgesics and antipyretics	0.5116	-0.0693	0.0180
J01F	Macrolides, Lincosamides, and streptogramins	0.4816	0.1198	-0.0282
N05B	Anxiolytics	0.4570	-0.1667	0.2307
S01A	Anti-infectives	0.4271	-0.0227	-0.2493
M01A	Anti-inflammatory and antirheumatic products, non-steroids	0.4174	0.1246	0.2261
D07A	Corticosteroids, plain	0.4097	0.0381	0.0199
D01A	Antifungals for topical use	0.3684	-0.0414	-0.0364
A07C	Electrolytes with carbohydrates	0.3648	-0.0920	-0.0283
D06A	Antibiotics for topical use	0.3583	-0.0033	0.0407
R06A	Antihistamines for systemic use	0.3299	0.6105	-0.1196
A03F	Propulsives	0.2802	0.0087	0.1934
SKN02	Dermatitis and eczema	0.2502	0.0842	-0.0067
ALL03	Allergic rhinitis	-0.1333	0.7546	0.0610
S01G	Decongestants and antiallergics	0.0216	0.7419	-0.0204
R01A	Decongestants and other nasal preparations for topical use	0.0808	0.6744	0.1262
ASMA	Asthma	0.1097	0.3489	0.0096
N03A	Antiepileptics	0.2292	-0.2209	0.6693
N06B	Psychostimulants, agents used for ADHD and nootropics	-0.1005	-0.0708	0.5403
NUR19	Developmental disorder	0.0843	-0.1170	0.3793
A02B	Drugs for peptic ulcers and GORD	0.1688	0.0530	0.3761
PSY14	Psychosocial disorders of childhood	-0.0763	0.1069	0.3287
EYE02	Blindness	-0.1745	0.1384	0.2778
NUT03	Obesity	-0.0570	0.0625	0.2760
EAR08	Deafness, hearing loss	0.0116	0.0680	0.2648
END05	Other endocrine disorders	-0.1296	0.0765	0.2309
EAR09	Chronic pharyngitis and tonsillitis	0.0115	0.1781	0.2292
MUS06	Kyphoscoliosis	-0.2194	0.1580	0.2269
CAR11	Disorders of lipid metabolism	-0.0803	0.1443	0.2166
SKN13	Disease of hair and hair follicles	-0.0241	0.1010	0.2163
MUS11	Congenital anomalies of limbs, hands, and feet	0.0419	0.0041	0.1473
S02C	Corticosteroids and antiinfectives in combination (Otologicals)	0.2499	0.1144	0.0886
S02A	Antiinfectives (Otologicals)	0.2308	0.0352	0.0700
PSY19	Sleep disorders of nonorganic origin	0.0974	0.1049	0.0517

Abbreviations: ADHD, attention deficit hyperactivity disorder; ATC, Anatomical Therapeutic Chemical Classification; EDC, Expanded Diagnostic Clusters; GORD, gastro-esophageal reflux disease.
Notes: Kaiser-Meyer-Olkin (KMO): 0.732; % cumulative variance: 33.2%.

S1E Table - Factor scores for women aged 15-44 years

ATC/EDC	Drug/Disease	Factor1	Factor2	Factor3
N06A	Antidepressants	0.8600	-0.0982	-0.1231
N03A	Antiepileptics	0.7610	-0.0769	-0.0670
N05B	Anxiolytics	0.7584	0.0082	-0.0801
N05A	Antipsychotics	0.5738	-0.0693	-0.0335
PSY09	Depression	0.5535	-0.0904	-0.0440
A02B	Drugs for peptic ulcers and GORD	0.4688	0.1871	0.1702
N02A	Opioids	0.4575	0.1610	-0.0062
PSY01	Anxiety, neuroses	0.4333	-0.0198	-0.0466
PSY19	Sleep disorders of nonorganic origin	0.3776	0.0054	0.0055
N02C	Antimigraine preparations	0.3742	0.0777	-0.0555
NUR21	Neurologic disorders, other	0.3556	-0.0168	0.0348
M01A	Anti-inflammatory and antirheumatic products, non-steroids	0.3550	0.3224	0.0806
NUR03	Peripheral neuropathy, neuritis	0.3093	0.0414	0.0350
A03F	Propulsives	0.2838	0.1342	0.0712
NUT03	Obesity	0.2665	0.0575	0.0821
R06A	Antihistamines for systemic use	-0.0568	0.8167	-0.0570
R03A	Adrenergics, inhalants	0.0513	0.7087	-0.1275
R01A	Decongestants and other nasal reparations for topical use	-0.0524	0.6800	-0.0444
S01G	Decongestants and antiallergics	-0.1184	0.6329	-0.0283
ASMA	Asthma	0.0009	0.4935	-0.1258
RES02	Acute lower respiratory tract infection	0.1409	0.4617	-0.0401
ALL03	Allergic rhinitis	-0.0826	0.4243	-0.0423
H02A	Corticosteroids for systemic use, plain	0.2442	0.4065	0.0103
J01F	Macrolides, lincosamides and streptogramins	0.1226	0.3837	0.0038
J01C	Beta-lactam antibacterials, penicillins	0.0880	0.3651	0.1273
J01M	Quinolone antibacterials	0.1836	0.3413	0.0686
J01D	Other beta-lactam antibacterials	0.1056	0.3320	0.0694
N02B	Other analgesics and antipyretics	0.2171	0.3169	0.2167
D07A	Corticoesteroids, plain	0.0221	0.3086	0.1492
B03A	Iron preparations	-0.0987	-0.0385	0.7959
H03C	Iodine therapy	-0.3295	-0.0731	0.6469
HEM02	Iron deficiency, other deficiency anemias	0.0079	-0.0121	0.5369
B03B	Vitamin B12 and folic acid	0.0477	0.0041	0.4798
H03A	Thyroid preparations	0.1296	-0.0427	0.4306
END04	Hypothyroidism	0.1069	-0.0596	0.3658
G01A	Antiinfectives and antiseptics, excl. Combinations with corticosteroids	0.0523	0.2204	0.2573
DIAB	Diabetes	0.0474	-0.0552	0.2416
B01A	Antithrombotic agents	0.1694	0.0756	0.2415
J01X	Other antibacterials	0.0715	0.1477	0.2121
D01A	Antifungicals for topical use	0.0352	0.2112	0.1952
G03A	Hormonal contraceptives for systemic use	-0.0845	0.1766	0.1879
END05	Other endocrine disorders	0.1306	-0.0026	0.1642
MUS14	Low back pain	0.2346	0.0541	0.1580
GSU08	Varicose veins of lower extremities	0.1783	0.0385	0.1106

CAR11	Disorders of lipid metabolism	0.2404	-0.0073	0.0933
SKN13	Disease of hair and hair follicles	0.0473	0.0580	0.0913
SKN02	Dermatitis and eczema	-0.0009	0.2372	0.0697
S01C	Antiinflamatory agents and antiinfectives in combination	0.0351	0.2309	0.0539
GSU06	Chronic cystic disease of the breast	0.0967	0.0026	0.0245
EAR08	Deafness, hearing loss	0.1316	0.0434	0.0023
EYE02	Blindness	0.0138	0.0912	-0.0090
MUS06	Kyphoscoliosis	-0.0162	0.0996	-0.0657

Abbreviations: ATC, Anatomical Therapeutic Chemical Classification; EDC, Expanded Diagnostic Clusters; GORD, gastro-esophageal reflux disease.

Notes: Kaiser-Meyer-Olkin (KMO): 0.740; % cumulative variance: 35.6%.

S1F Table- Factor scores for women aged 45-65 years

ATC/EDC	Drug/Disease	Factor1	Factor2	Factor3	Factor4
N06A	Antidepressants	0.8980	-0.1147	-0.0025	-0.1195
N05B	Anxiolytics	0.6682	0.0152	-0.0202	-0.0019
PSY09	Depression	0.6131	-0.0878	0.0057	-0.0435
N05C	Hypnotics and sedatives	0.5592	-0.0438	-0.0072	0.0568
N03A	Antiepileptics	0.5406	0.0020	0.0741	0.0453
PSY01	Anxiety, neuroses	0.4116	-0.0001	-0.0337	-0.0995
N02A	Opioids	0.3805	0.1373	0.1475	0.1399
PSY19	Sleep disorders of nonorganic origin	0.3618	-0.0079	-0.0166	0.0931
A02B	Drugs for peptic ulcers and GORD	0.3379	0.2092	0.2092	0.2417
M01A	Antiinflamatory and antirheumatic products, non-steroids	0.2788	0.2881	-0,0121	0,2077
R03A	Adrenergics, inhalants	-0.1009	0.7548	0.1040	-0,1105
R06A	Antihistamines for systemic se	-0.0746	0.7487	-0.0394	-0,0587
R01A	Decongestants and other nasal preparations for topical use	-0.0788	0.6301	-0.0702	-0.0300
ASMA	Asthma	-0.0894	0.5872	0.0567	-0.1262
H02A	Corticosteroids for systemic use, pain	0.0475	0.4867	0.0238	0.1173
J01F	Macrolides, lincosamides and streptogramins	0.0063	0.4468	0.0225	0.0494
J01M	Quinolone antibacterials	0.0253	0.4313	0.0749	0.0600
ALL03	Allergic rhinitis	-0.0102	0.4032	-0.1253	-0.0428
J01C	Beta-lactam antibacterials, penicillins	0.0426	0.3853	0.0700	0.0282
N02B	Other analgesics and antipyretics	0.1672	0.3269	0.1711	0.1550
HTA	Hypertension	-0.1308	-0.0771	0.9601	-0.0185
C09A	Ace inhibitors, plain	-0.1033	-0.0504	0.7041	-0.0338
DIAB	Diabetes	0.0166	-0.0071	0.5854	-0.0658
NUT03	Obesity	0.0607	0.0899	0.5014	-0.0963
B01A	Antithrombotic agents	0.0852	0.0688	0.3699	0.1291
CAR11	Disorders of lipid metabolism	0.0637	-0.0408	0.2951	0.1722
A12A	Calcium	-0.1196	-0.0178	-0.0442	0.8032
END02	Osteoporosis	-0.1011	-0.0544	-0.0526	0.7869
D07A	Corticosteroids, plain	0.0506	0.2863	0.0381	0.0580
ARTRITIS	Arthritis	0.0891	0.0818	0.1864	0.2411
CANCER	Cancer	0.0471	-0.0013	0.0264	0.1828
GSU08	Varicose veins of lower extremities	0.0318	0.1295	0.0818	0.1441
END05	Other endocrine disorders	0.0507	0.0433	0.0539	0.1128
MUS14	Low back pain	0.2051	0.1235	-0.0322	0.1092
EAR08	Deafness, hearing loss	0.0635	0.0906	0.0211	0.0837
END04	Hypothyroidism	0.0776	0.0098	0.0928	0.0771
J01X	Otros antibacterianos	0.0900	0.1560	0.0389	0.0743
NUR21	Neurologic disorders, other	0.1922	0.0659	0.0580	0.0658
EYE02	Blindness	0.0680	0.0847	-0.0031	0.0607
SKN13	Disease of hair and hair follicles	0.0614	0.1021	-0.0415	0.0529
NUR03	Peripheral neuropathy, neuritis	0.1800	0.0977	0.0773	0.0470
SKN02	Dermatitis and eczema	0.0487	0.2127	0.0134	0.0406
HEM02	Iron deficiency, other deficiency anemias	0.1139	0.0882	0.0526	-0.0781

Abbreviations: ATC, Anatomical Therapeutic Chemical Classification; EDC, Expanded Diagnostic Clusters; GORD, gastro-esophageal reflux disease.
Notes: Kaiser-Meyer-Olkin (KMO): 0.803; % cumulative variance: 31.3%.
